# Suicidality and Quality of Life in Treatment-Resistant Depression Patients in Latin America: Secondary Interim Analysis of the TRAL Study

**DOI:** 10.3389/fpsyt.2022.812938

**Published:** 2022-03-02

**Authors:** Ricardo Corral, Hernan Alessandria, Lina María Agudelo Baena, Eugenio Ferro, Xochitl Duque, Lucas Quarantini, Marco Antonio Caldieraro, Patricia Cabrera, Gabriela Kanevsky

**Affiliations:** ^1^Fundación para el Estudio y Tratamiento de las Enfermedades Mentales, Buenos Aires, Argentina; ^2^Clinica Privada de Salud Mental Santa Teresa de Avila, Buenos Aires, Argentina; ^3^Centro de Investigaciones de la E.S.E. Hospital Mental de Antioquia, Bello-Antioquia, Colombia; ^4^Instituto Colombiano del Sistema Nervioso – Clínica Montserrat, Bogotá, Colombia; ^5^Institute for Social Security and Services for State Workers (ISSSTE), Mexico City, Mexico; ^6^Hospital das Clínicas de Salvador, Salvador, Brazil; ^7^Hospital de Clínicas de Porto Alegre, Porto Alegre, Brazil; ^8^Janssen-Cilag-Colombia, Bogotá, Colombia; ^9^Janssen-Cilag-Argentina, Buenos Aires, Argentina

**Keywords:** treatment-resistant depression, major depressive disorder, suicidal ideation, quality of life, Latin America

## Abstract

**Background:**

A large proportion of patients with major depressive disorder (MDD) have treatment-resistant depression (TRD). The TRAL study examines the impact of TRD on suicidality and health-related quality of life (HRQoL) among MDD patients in 4 Latin American countries.

**Methods:**

In this multicenter, prospective, observational study, MDD patients were recruited from 33 sites in Mexico, Colombia, Brazil, and Argentina. Patients were assessed for TRD, defined as failure to respond to ≥2 antidepressant medications of adequate dose and duration. Other assessments included current disease status, Mini International Neuropsychiatric Interview (MINI), Columbia-Suicide Severity Rating Scale (C-SSRS), 5 Level EQ-5D (EQ-5D-5L), Patient Health Questionnaire-9 (PHQ-9), and Sheehan Disability Scale (SDS).

**Results:**

1,475 MDD patients were included in the analysis (mean age, 45.6 years; 78% women), and 429 met criteria for TRD. Thoughts of suicide and suicide attempts were more common among TRD patients (38.7%) compared with non-TRD patients (24.9%; *P* < 0.0001), according to the current disease status questionnaire. The C-SSRS showed that lifetime suicidal behavior was significantly more common among TRD patients than non-TRD patients (13.8 vs. 10.0%; *P* = 0.0384). Compared with non-TRD patients, TRD patients showed significantly greater adverse impacts on QoL (EQ-5D-5L), more severe depression (PHQ-9), and greater functional impairment (SDS).

**Conclusion:**

TRD patients in clinical sites from Mexico, Colombia, Brazil, and Argentina were more likely to experience suicidality and negative effects on HRQoL than non-TRD patients.

## Introduction

Major depressive disorder (MDD), which affects >300 million individuals worldwide, can severely impact functionality and quality of life (QoL) and may even be fatal (1, 2). Suicide is the cause of death for >800,000 individuals worldwide each year (3), and MDD patients are at increased risk for suicide (4). Compared with other psychiatric diagnoses, MDD and other depression-related diagnoses are associated with suicidal behavior and suicide at a higher rate (3, 5–7). According to suicide mortality data from 8 health care systems in the Mental Health Research Network, among those patients with mental health conditions who died by suicide, ~80% had depression; the risk of mortality from a suicide attempt was 7-fold greater in those with depression in comparison to those without depression in the general population (8). The presence of depression in the past year has been found to increase the risk of a suicide attempt 8-fold, and recent suicide attempts are significantly more common in those with a depressive disorder than in those without a depressive disorder after controlling for demographic factors (9). Increases in depression severity are associated with an increased risk of suicidal ideation and suicide attempts (10, 11). In one study, those with severe depression were 20 times more likely than those with no or mild depression to report suicidal ideation (10). In another study, those with the most severe depression symptoms had an 8-fold increased risk of a suicide attempt (11). Suicidal ideation and behavior can present significant treatment challenges, and identification of risk factors for suicide, including depression severity, is an important aspect of appropriate management of MDD patients (12). Recent work examining the genetic, physiological, and neurobiological underpinnings of suicidal ideation has provided some insight into relevant neurological pathways and identified potentially relevant biomarkers, but identifying at-risk patients remains a critical challenge (12, 13).

Approximately half of MDD patients do not respond to a first-line treatment; of those who continue to a second treatment, a further two-thirds do not respond (14). Treatment-resistant depression (TRD) is defined as failure to respond to ≥2 antidepressant regimens of adequate dose and duration (15, 16). Compared to treatment-responsive MDD patients (non-TRD), TRD patients have shown both increased suicidality (17) and lower health-related QoL (HRQoL) (17–19). The prevalence and burden of TRD in Latin America are not well documented, and more research is needed to understand the impact of MDD and TRD on suicidality and HRQoL in regions outside the United States and Europe. In Latin America, access to mental health care is more difficult, and gaps in access to care are more pronounced compared with the United States (20).

The TRAL study is a multicenter, prospective, observational, non-interventional study of TRD in 4 Latin American countries: Mexico, Colombia, Argentina, and Brazil. The study includes patients receiving care from clinical centers that treat mental disorders; it is not a population-based study. The primary objective of this study was to estimate the prevalence of TRD among MDD patients being treated in a psychiatry reference site. The secondary objectives were to describe the characteristics of MDD patients, including comorbidities, treatment standards, severity of symptoms, and level of disability, and to evaluate suicidality risk (ideation and attempts) in TRD patients. The primary interim analysis of the first phase of the TRAL study revealed that 29% of MDD patients had TRD (21). Regional prevalence of TRD among MDD patients was as follows: 21% in Mexico, 32% in Colombia, 33% in Argentina, and 40% in Brazil. Results of the analysis showed that suicidality, anxiety, and work impairment were significantly greater among TRD patients than non-TRD patients (21). Here we report the secondary interim results from the first phase of the TRAL study, including detailed measures of suicidality, HRQoL, and related factors.

## Materials and Methods

### Objectives

Objectives of the current analyses were to evaluate suicidal ideation, behavior, and related measures among TRD patients in comparison with non-TRD MDD patients being treated in a psychiatric reference site (clinic, ambulatory, hospital, day-hospital) in 4 Latin American countries (Mexico, Colombia, Brazil, and Argentina), and to evaluate HRQoL and related measures among TRD patients vs. non-TRD MDD patients.

### Study Design and Population

This was a multicenter, prospective, observational, non-interventional study consisting of a screening phase, cross-sectional evaluation of demographic, symptom, suicidality, HRQoL, and functionality data (Phase 1); and 1-year follow up of the subset of TRD patients (Phase 2). Detailed study design methods have been described in the primary report and are briefly summarized here. Included patients were adult women and men (aged ≥18 years) with a diagnosis of MDD per the *Diagnostic and Statistical Manual of Mental Disorders, 5th Edition* and confirmed by the Mini-International Neuropsychiatric Interview, version 7.0.2 (MINI). Patients had a new or continued episode of depression (treated or untreated) at the time of enrollment and were capable of completing study assessments. Diagnosis of TRD was based on the following criteria: treatment and adequate follow-up with ≥2 antidepressants, and lack of complete response to treatment based on the Montgomery-Asberg Depression Rating Scale (MADRS), where response was defined as 50% improvement in MADRS total score. MADRS is a 10-item depression scale designed to be responsive to treatment effects (22). Key exclusion criteria consisted of diagnosis of psychosis, schizophrenia, bipolar disorder, schizoaffective disorder, or dementia; serious substance dependence (as determined by the investigator); and current participation in another clinical study.

Data sources for the study were patient medical records, questionnaires, scales, and assessments completed by patients and investigators as part of the study.

### Analyses

Suicidality and HRQoL variables were assessed among all MDD patients (TRD and non-TRD). These variables included suicidal ideation, behavior, and related measures, as assessed by current disease status (a questionnaire regarding the patient's diagnosis, symptoms, and treatment), MINI (evaluated according to standards in which the presence or absence of suicidal thoughts and suicide attempts as well as feelings such as “hopelessness/pessimism” and “guilt/worthlessness/helplessness” were noted), and the Columbia-Suicide Severity Rating Scale (C-SSRS). Of note, on the C-SSRS suicidal ideation scale, only patients who answered “yes” to item 2 of the ideation scale (non-specific active suicidal thoughts [lifetime]) were asked subsequent questions on the ideation scale. Therefore, responses to items 3 through 5 of the scale are represented as percentages of the total number of patients responding “yes” to item 2 (lifetime) rather than as percentages of all respondents. HRQoL and related measures were assessed using the 5 Level EQ-5D (EQ-5D-5L; a health status questionnaire that provides a simple profile and an index value) (23), Patient Health Questionnaire-9 (PHQ-9; a measure of depression severity) (24), and Sheehan Disability Scale (SDS; a measure of disability and functional impairment) (25).

### Statistical Analyses

Descriptive statistics were used to evaluate variables in all MDD patients and by group (non-TRD and TRD). Analysis of quantitative variables included calculation of mean, standard deviation (SD), or median and interquartile range (IQR) depending on the normal distribution of each variable. Analysis of qualitative variables included calculation of frequencies and percentages. Comparisons between groups for categorical variables were performed using the chi square test (CS) or Fisher Exact test. Comparisons between groups for quantitative variables were performed using the *t*-test for independent samples or the Mann-Whitney non-parametric test (MW), according to the assumption validations of the statistical tests. Partial dates were completed by investigators in a standardized fashion; no other imputation of missing data was performed.

## Results

### Study Population and TRD Prevalence

Detailed prevalence information has been described in the primary report and is briefly summarized here. A total of 1,544 patients were screened and 1,475 (96%) were included in the Phase 1 analysis dataset. Patient data were collected from 33 centers in 4 countries: Mexico (*n* = 697; 47% of all patients), Colombia (*n* = 162; 11%), Brazil (*n* = 396; 27%), and Argentina (*n* = 220; 15%). In total, 429 patients had TRD [29% of all MDD patients; 95% confidence interval (CI), 27–31%]. Sociodemographic and clinical characteristics of all MDD patients have been reported previously (21).

### Suicidality and Related Factors in Patients With Non-TRD and TRD

A comparison of suicidal ideation and behavior measures across scales is provided in Table 1. Among MDD patients, 28.9% had thoughts of suicide or suicide attempts, as measured by the current disease status questionnaire. Thoughts of suicide and suicide attempts were more common among TRD patients (38.7%) in comparison with non-TRD patients (24.9%; *P* < 0.0001). The questionnaire also allowed assessment of feelings such as “hopelessness and/or pessimism” and “guilt, worthlessness, and/or helplessness”; these were reported in 80.8 and 77.9% of MDD patients, respectively. TRD patients, vs. non-TRD patients, more frequently reported both hopelessness/pessimism (90.7 vs. 76.8%; CS, *P* < 0.0001) and guilt/worthlessness/helplessness (83.0 vs. 75.8%; CS, *P* = 0.0026).

**Table 1:** Suicidal ideation and behavior across scales.

	**All MDD (*N* = 1,475)**	**Non-TRD (*n* = 1,046)**	**TRD (*n* = 429)**	***P-*value (non-TRD vs. TRD)**
**Current disease status**				
Thoughts of suicide, suicide attempts, *n* (%)	426 (28.9%)	260 (24.9%)	166 (38.7%)	CS: <0.0001
**MINI, meets criteria**, ***n*** **(%)**				
Current suicidality (past month)	358 (24.3%)	207 (19.8%)	151 (35.2%)	CS: <0.0001
Lifetime attempt	265 (18.0%)	143 (13.7%)	122 (28.4%)	CS: <0.0001
Current suicidal behavior disorder	96 (6.5%)	59 (5.6%)	37 (8.6%)	CS: 0.0349
**C-SSRS**				
Suicidal ideation score^*^				MW: 0.9144
*n*	512	308	204	
Median	4.00	4.00	4.00	
Q1	3.00	3.00	3.00	
Q3	5.00	5.00	5.00	
IQR	2.00	2.00	2.00	
Suicidal behavior score^†^	356 (24.2%)	215 (20.6%)	141 (32.9%)	CS: <0.0001

*MDD, major depressive disorder; TRD, treatment-resistant depression; CS, chi square test; MINI, Mini-International Neuropsychiatric Interview, version 7.0.2; C-SSRS, Columbia-Suicide Severity Rating Scale; MW, Mann-Whitney non-parametric test; Q, quartile; IQR, interquartile range*.

* *Maximum suicidal ideation category (range: 1–5; 1 = wish to be dead; 2 = non-specific active suicidal thoughts; 3 = active suicidal ideation with any methods [not plan], without intent to act; 4 = active suicidal ideation with some intent to act, without specific plan; 5 = active suicidal ideation with specific plan and intent)*.

† *A “yes” answer to 1 of the 5 suicidal behavior questions*.

According to results from the MINI, 24.3% of MDD patients met the criteria for current suicidality (score of ≥1 on the suicidality subscale) and 18.0% reported a lifetime suicide attempt. Both current suicidality and lifetime suicide attempts were significantly more common in TRD patients than in non-TRD patients (current, 35.2 vs. 19.8%; lifetime, 28.4 vs. 13.7%; both *P* < 0.0001). Among all MDD patients, 6.5% met the criteria for suicidal behavior disorder [defined as presence of a suicide attempt within the past 2 years (1)] and 5.2% were considered “in early remission” from this disorder. Current suicidal behavior disorder was also more common among TRD patients than non-TRD patients (8.6 vs. 5.6%; *P* = 0.0349). Furthermore, 9.3% of TRD patients were considered in early remission from suicidal behavior disorder in comparison to 3.5% of non-TRD patients (*P* < 0.0001), indicating that TRD patients were more likely to have a recent suicidal behavior disorder.

There were no statistically significant differences between groups for suicidal behavior disorder as a current primary diagnosis, but significantly more TRD patients, vs. non-TRD patients, had a primary diagnosis of early remission suicidal behavior disorder (2.1 vs. 0.6%; *P* = 0.0175). TRD patients, vs. non-TRD patients, also reported significantly higher prevalence of comorbid generalized anxiety disorder (GAD; 26.3 vs. 17.7%; *P* = 0.0002), post-traumatic stress disorder (PTSD; 6.5 vs. 3.3%; *P* = 0.0061), and substance use disorder (non-alcohol; 2.1 vs. 0.6%; *P* = 0.0175). Differences between groups for primary diagnosis of GAD, PTSD, or substance use disorder were not significant.

**Figure 1:**
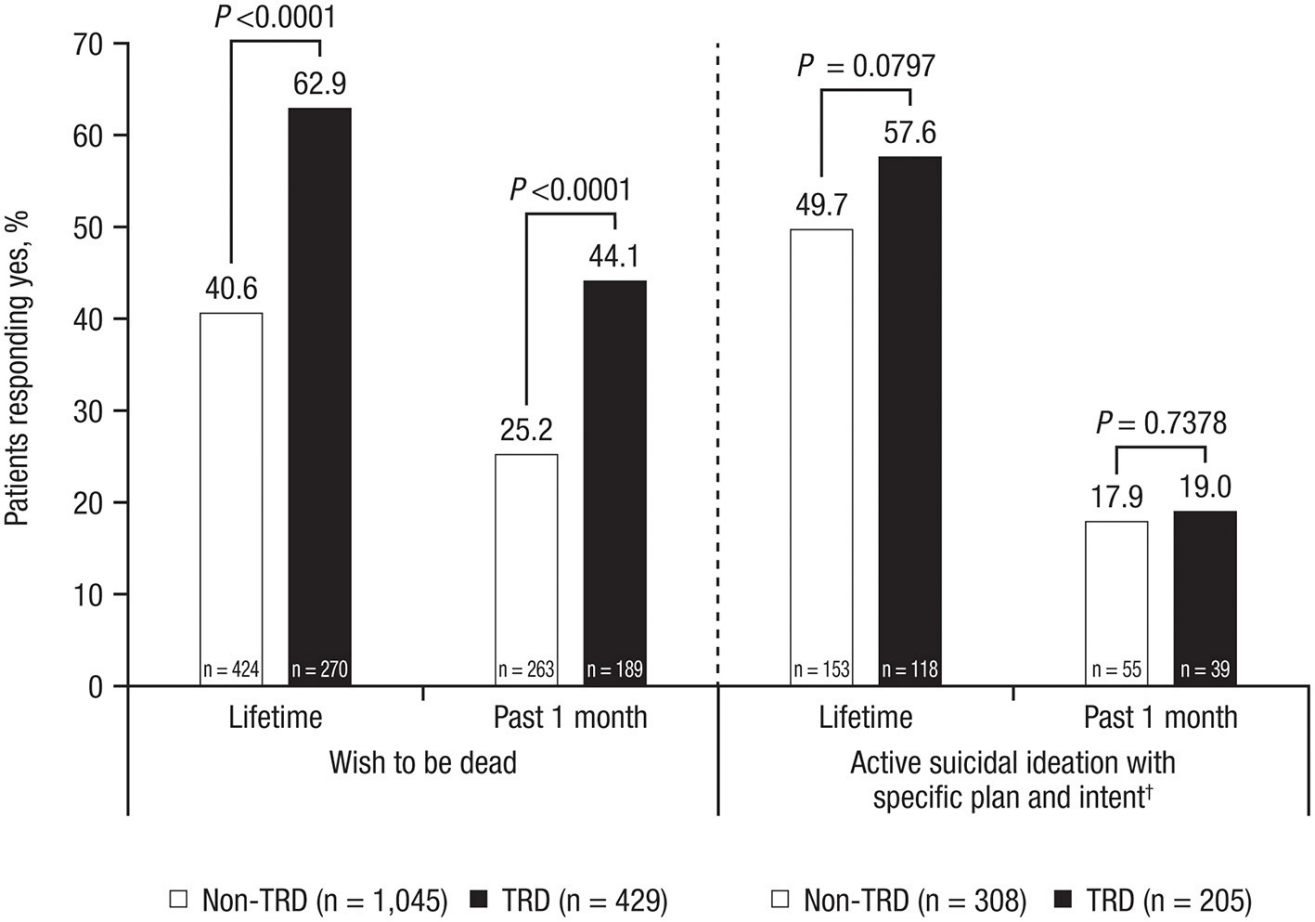
Suicidal ideation in TRD patients vs. non-TRD patients, evaluated with C-SSRS*. TRD, treatment-resistant depression; C-SSRS, Columbia-Suicide Severity Rating Scale. *P-values represent differences between groups as measured by the chi square test. ^†^Only patients who answered “yes” to having “non-specific active suicidal thoughts” were asked subsequent questions about intensity of ideation.

Among all MDD patients, the median suicidal ideation score on the C-SSRS was 4.0 (IQR, 3.0–5.0; Table 1). Over a lifetime, 47.1% of MDD patients had a “wish to be dead” (30.7% in the past 1 month; Table 2). Of the 513 MDD patients who reported at least non-specific active suicidal thoughts (lifetime), 52.8% had active suicidal ideation with specific plan and intent (18.3% in the past 1 month; Table 2). Median C-SSRS suicidal ideation score did not differ significantly between TRD patients and non-TRD patients (Table 1). However, TRD patients were more likely to have experienced a “wish to be dead” both over a lifetime (62.9 vs. 40.6%) and in the past month (44.1 vs. 25.2%; all *P* < 0.0001; Figure 1; Table 2).

**Table 2:** C-SSRS suicidal ideation and behavior scale.

	**All MDD (*N* = 1,475)**	**Non-TRD (*n* = 1,046)**	**TRD (*n* = 429)**	***P-*value (non-TRD vs. TRD)**
**Suicidal ideation (1–5)**, ***n*** (%)^*^				
1) Wish to be dead	452 (30.7%)	263 (25.2%)	189 (44.1%)	CS: <0.0001
2) Non-specific active suicidal thoughts	278 (18.9%)	171 (16.4%)	107 (24.9%)	CS: 0.0001
3) Active suicidal ideation with any methods (not plan), without intent to act	214 (41.7%)	127 (41.2%)	87 (42.4%)	CS: 0.7863
4) Active suicidal ideation with some intent to act, without specific plan	156 (30.5%)	96 (31.2%)	60 (29.4%)	CS: 0.6724
5) Active suicidal ideation with specific plan and intent	94 (18.3%)	55 (17.9%)	39 (19.0%)	CS: 0.7378
**Suicidal behavior (6–10), *n* (%)^†^**				
6) Preparatory acts or behavior	65 (4.4%)	38 (3.6%)	27 (6.3%)	CS: 0.0228
7) Aborted attempt	34 (2.3%)	25 (2.4%)	9 (2.1%)	CS: 0.7396
8) Interrupted attempt	41 (2.8%)	28 (2.7%)	13 (3.0%)	CS: 0.6993
9) Non-fatal suicide attempt	87 (5.9%)	61 (5.8%)	26 (6.1%)	CS: 0.8526
10) Completed suicide	NA	NA	NA	NA

*C-SSRS, Columbia-Suicide Severity Rating Scale; MDD, major depressive disorder; TRD, treatment-resistant depression; CS, chi square test; NA, not applicable*.

* *Past 1 month*.

† *Past 3 months*.

**Figure 2:**
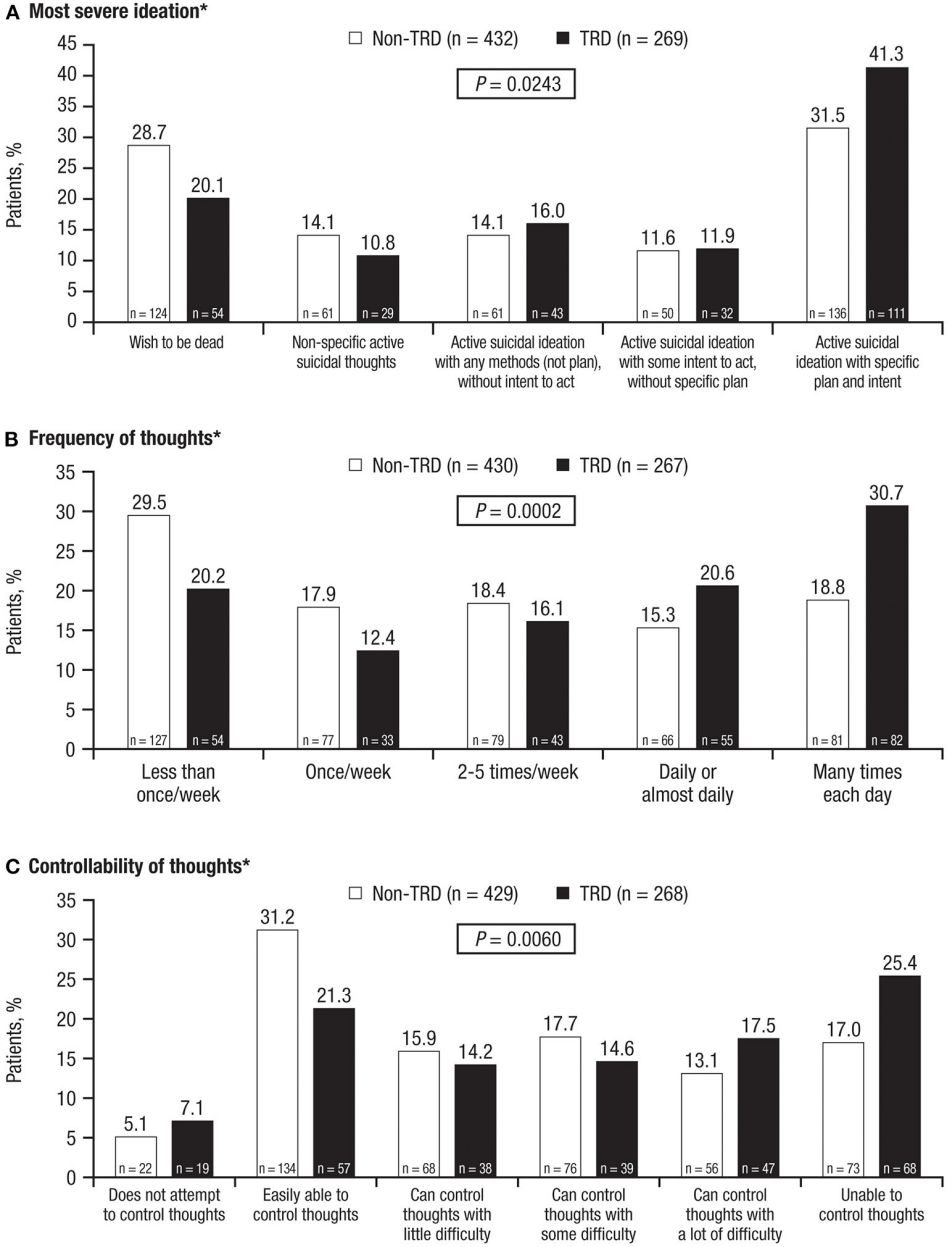
Lifetime categories of suicidal ideation, evaluated with C-SSRS. **(A)** Most severe ideation, **(B)** Frequency of thoughts, and **(C)** Controllability of thoughts. TRD, treatment-resistant depression; C-SSRS, Columbia-Suicide Severity Rating Scale. *P-values represent differences between groups as measured by the chi square test.

TRD patients were also numerically more likely than non-TRD patients to report lifetime active suicidal ideation with specific plan and intent (57.6 vs. 49.7%; *P* = 0.0797). TRD patients reported more severe lifetime suicidal ideation, with significantly higher scores for “most severe ideation” (Figure 2A; *P* = 0.0243), “frequency of thoughts” (Figure 2B; *P* = 0.0002), and “controllability of thoughts” (Figure 2C; *P* = 0.0060).

**Figure 3:**
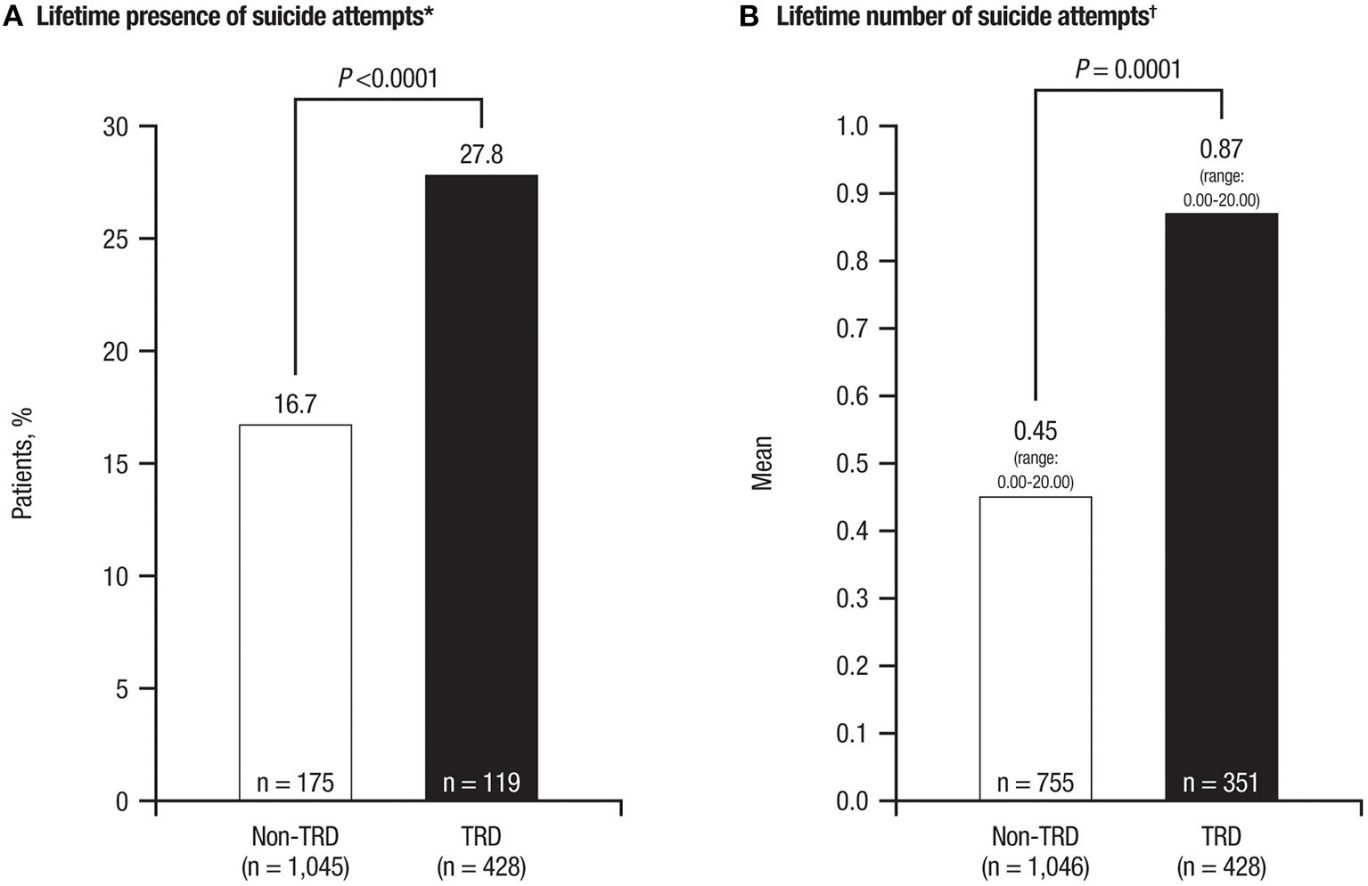
Lifetime suicidal behavior in TRD patients vs. non-TRD patients, evaluated with C-SSRS. **(A)** Lifetime presence of suicide attempts. **(B)** Lifetime number of suicide attempts. TRD, treatment-resistant depression; C-SSRS, Columbia-Suicide Severity Rating Scale. *P-value represents differences between groups as measured by the chi square test. ^†^*P*-value represents differences between groups as measured by the Mann-Whitney non-parametric test.

Among all MDD patients, 24.2% answered “yes” to ≥1 indicator of suicidal behavior, as measured by C-SSRS; a significantly greater proportion of TRD patients than non-TRD patients had ≥1 indicator of suicidal behavior (32.9 vs. 20.6%; *P* < 0.0001; Table 1). Among all MDD patients, 20.0% reported a lifetime suicide attempt, with a mean of 0.59 (range: 0.00–20.00) total attempts. Lifetime suicidal behavior was significantly more common among TRD patients than non-TRD patients (13.8 vs. 10.0%; *P* = 0.0384). TRD patients, vs. non-TRD patients, were more likely to have made a lifetime suicide attempt (27.8 vs. 16.7%; *P* < 0.0001) and to have a higher number of lifetime attempts [mean number (range) 0.87 (0.00–20.00) vs. 0.45 (0.00–20.00); *P* = 0.0001; Figure 3]. Moreover, suicide attempts in TRD patients were significantly more likely to be fatal than in non-TRD patients (Table 3).

**Table 3:** C-SSRS potential lethality of suicide attempts.

	**All MDD (*N* = 1,475)**	**Non-TRD (*n* = 1,046)**	**TRD (*n* = 429)**	***P-*value (non-TRD vs. TRD)**
**Potential lethality**				
**Most recent attempt**, ***n*** **(%)**				
0. Behavior not likely to result in injury	51 (55.4%)	40 (64.5%)	11 (36.7%)	CS: 0.0187
1. Behavior likely to result in injury but not likely to cause death	10 (10.9%)	7 (11.3%)	3 (10.0%)	
2. Behavior likely to result in death despite available medical care	31 (33.7%)	15 (24.2%)	16 (53.3%)	
Total	92	62	30	
**Most lethal attempt**, ***n*** **(%)**				
0. Behavior not likely to result in injury	49 (57.0%)	39 (67.2%)	10 (35.7%)	CS: 0.0115
1. Behavior likely to result in injury but not likely to cause death	9 (10.5%)	6 (10.3%)	3 (10.7%)	
2. Behavior likely to result in death despite available medical care	28 (32.6%)	13 (22.4%)	15 (53.6%)	
Total	86	58	28	
**Initial/first attempt**, ***n*** **(%)**				
0. Behavior not likely to result in injury	52 (55.9%)	40 (62.5%)	12 (41.4%)	CS: 0.0563
1. Behavior likely to result in injury but not likely to cause death	12 (12.9%)	9 (14.1%)	3 (10.3%)	
2. Behavior likely to result in death despite available medical care	29 (31.2%)	15 (23.4%)	14 (48.3%)	
Total	93	64	29	

*C-SSRS, Columbia-Suicide Severity Rating Scale; MDD, major depressive disorder; TRD, treatment-resistant depression; CS, chi square test*.

### HRQoL and Related Measures in Patients With TRD and Non-TRD

Results from the EQ-5D-5L HRQoL questionnaire demonstrated that TRD patients in comparison to non-TRD patients were significantly more impacted on the mobility, self-care, usual activities, pain/discomfort, anxiety/depression, and current health subscales (all *P* < 0.0001; Table 4). Among non-TRD patients, 66.4% reported no or slight pain or discomfort vs. 47.6% of TRD patients; in contrast, 23.5% of TRD patients reported severe or extreme pain or discomfort in comparison to 10.9% of non-TRD patients. Of non-TRD patients, 11.6% reported no feelings of anxiety or depression vs. 2.8% of TRD patients; 26.3% of non-TRD patients reported feeling severely or extremely anxious or depressed in comparison with 43.1% of TRD patients.

**Table 4:** EQ-5D-5L HRQoL questionnaire.

	**All MDD (*N* = 1,475)**	**Non-TRD (*n* = 1,046)**	**TRD (*n* = 429)**	***P-*value (non-TRD vs. TRD)**
**Mobility**, ***n*** **(%)**				
I have no problems walking	954 (64.7%)	712 (68.1%)	242 (56.4%)	CS: <0.0001
I have slight problems walking	312 (21.2%)	231 (22.1%)	81 (18.9%)	
I have moderate problems walking	161 (10.9%)	81 (7.8%)	80 (18.6%)	
I have severe problems walking	42 (2.8%)	17 (1.6%)	25 (5.8%)	
I am unable to walk	5 (0.3%)	4 (0.4%)	1 (0.2%)	
Total	1,474	1,045	429	
**Self-care**, ***n*** **(%)**				
I have no problems washing or dressing myself	938 (63.6%)	697 (66.7%)	241 (56.2%)	CS: <0.0001
I have slight problems washing or dressing myself	320 (21.7%)	233 (22.3%)	87 (20.3%)	
I have moderate problems washing or dressing myself	162 (11.0%)	88 (8.4%)	74 (17.2%)	
I have severe problems washing or dressing myself	49 (3.3%)	22 (2.1%)	27 (6.3%)	
I am unable to wash or dress myself	5 (0.3%)	5 (0.5%)	0	
Total	1,474	1,045	429	
**Usual activities**, ***n*** **(%)**				
I have no problems doing my usual activities	375 (25.4%)	316 (30.2%)	59 (13.8%)	CS: <0.0001
I have slight problems doing my usual activities	435 (29.5%)	338 (32.3%)	97 (22.6%)	
I have moderate problems doing my usual activities	458 (31.1%)	275 (26.3%)	183 (42.7%)	
I have severe problems doing my usual activities	169 (11.5%)	96 (9.2%)	73 (17.0%)	
I am unable to do my usual activities	37 (2.5%)	20 (1.9%)	17 (4.0%)	
Total	1,474	1,045	429	
**Pain/discomfort**, ***n*** **(%)**				
I have no pain or discomfort	445 (30.2%)	346 (33.1%)	99 (23.1%)	CS: <0.0001
I have slight pain or discomfort	453 (30.7%)	348 (33.3%)	105 (24.5%)	
I have moderate pain or discomfort	361 (24.5%)	237 (22.7%)	124 (28.9%)	
I have severe pain or discomfort	182 (12.3%)	99 (9.5%)	83 (19.3%)	
I have extreme pain or discomfort	33 (2.2%)	15 (1.4%)	18 (4.2%)	
Total	1,474	1,045	429	
**Anxiety/depression**, ***n*** **(%)**				
I am not anxious or depressed	133 (9.0%)	121 (11.6%)	12 (2.8%)	CS: <0.0001
I am slightly anxious or depressed	338 (22.9%)	280 (26.8%)	58 (13.5%)	
I am moderately anxious or depressed	543 (36.8%)	369 (35.3%)	174 (40.6%)	
I am severely anxious or depressed	339 (23.0%)	206 (19.7%)	133 (31.0%)	
I am extremely anxious or depressed	121 (8.2%)	69 (6.6%)	52 (12.1%)	
Total	1,474	1,045	429	
Health in the current day^*^				
*n*	1,473	1,044	429	MW: <0.0001
Median	60.00	65.00	50.00	
Q1	50.00	50.00	40.00	
Q3	75.00	79.00	65.00	
IQR	25.00	29.00	25.00	

*HRQoL, health-related quality of life; MDD, major depressive disorder; TRD, treatment-resistant depression; CS, chi square test; MW, Mann-Whitney non-parametric test; Q, quartile; IQR, interquartile range*.

* *Assessed using a visual analog scale ranging from 0 (worst health) to 100 (best health)*.

**Figure 4:**
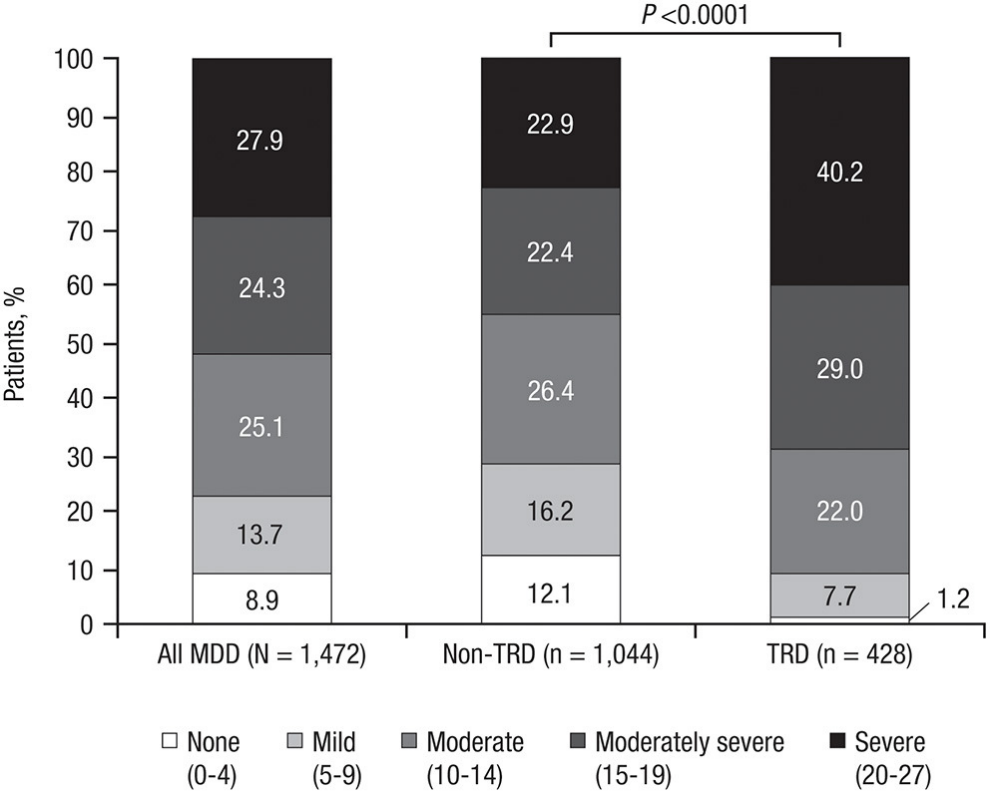
Depression severity on the PHQ-9*. PHQ-9, Patient Health Questionnaire-9; MDD, major depressive disorder; TRD, treatment-resistant depression. *P-value represents chi square test.

Depression severity scores on the PHQ-9 were higher in TRD patients (*P* < 0.0001), with 40.2% of TRD patients characterized as having severe depression (vs. 22.9% of non-TRD patients; Figure 4). Approximately two-thirds of TRD patients (vs. less than half of non-TRD patients) reported facing problems that made it “very difficult” or “extremely difficult” to do work, take care of things at home, or get along with other people (Table 5).

**Table 5:** Selected items from the PHQ-9.

	**All MDD (*N* = 1,475)**	**Non-TRD (*n* = 1,046)**	**TRD (*n* = 429)**	***P-*value (non-TRD vs. TRD)**
Total score^*^				
*n*	1,472	1,044	428	MW: <0.0001
Median	15.00	14.00	18.00	
Q1	10.00	9.00	13.00	
Q3	20.00	19.00	22.00	
IQR	10.00	10.00	9.00	
**If you checked off any problems, how difficult have these problems made it for you to do your work, take care of things at home, or get along with other people?**, ***n*** **(%)**				
Not difficult at all	152 (10.3%)	145 (13.9%)	7 (1.6%)	CS: <0.0001
Somewhat difficult	571 (38.8%)	437 (41.9%)	134 (31.3%)	
Very difficult	618 (42.0%)	392 (37.5%)	226 (52.8%)	
Extremely difficult	131 (8.9%)	70 (6.7%)	61 (14.3%)	
Total	1,472	1,044	428	

*PHQ-9, Patient Health Questionnaire-9; MDD, major depressive disorder; TRD, treatment-resistant depression; MW, Mann-Whitney non-parametric test; Q, quartile; IQR, interquartile range; CS, chi square test*.

* *Total score ranges from 0 to 27; higher values indicate higher depression severity*.

On the SDS, TRD patients reported higher levels of disruption to work/school, social life/leisure activities, and family/home responsibilities than did non-TRD patients (all *P* < 0.0001; Table 6). Of TRD patients, 17.4% responded that symptoms had extremely disrupted work/school (vs. 7.6% of non-TRD patients), 17.5% reported extremely disrupted social life/leisure activities (vs. 7.6% of non TRD patients), and 11.0% reported extremely disrupted family life/home responsibilities (vs. 6.0% of non-TRD patients). TRD patients also had higher median total SDS scores than did non-TRD patients [21.0 (IQR, 16.0–26.0) vs. 16.0 (IQR, 11.0–21.0); *P* < 0.0001]. TRD patients reported a greater median (IQR) number of days in the previous 7 days that they were unable to carry out normal daily responsibilities [1.00 (0.00–4.00) vs. 0.00 (0.00–2.00); *P* < 0.0001].

**Table 6:** SDS questionnaire.

	**All MDD (*N* = 1,475)**	**Non-TRD (*n* = 1,046)**	**TRD (*n* = 429)**	***P-*value (non-TRD vs. TRD)**
The symptoms have disrupted your work/school^*^				
*n*	1,134	794	340	MW: <0.0001
Median	6.00	5.00	7.00	
Q1	4.00	3.00	5.00	
Q3	8.00	7.00	9.00	
IQR	4.00	4.00	4.00	
The symptoms have disrupted your social life/leisure activities^*^				
*n*	1,473	1,044	429	MW: <0.0001
Median	6.00	5.00	7.00	
Q1	4.00	3.00	5.00	
Q3	8.00	7.00	9.00	
IQR	4.00	4.00	4.00	
The symptoms have disrupted your family life/home responsibilities^*^				
*n*	1,473	1,044	429	MW: <0.0001
Median	6.00	5.00	7.00	
Q1	4.00	3.00	5.00	
Q3	8.00	7.00	8.00	
IQR	4.00	4.00	3.00	
**Total score**				
*n*	1,134	794	340	MW: <0.0001
Median	18.00	16.00	21.00	
Q1	12.00	11.00	16.00	
Q3	23.00	21.00	26.00	
IQR	11.00	10.00	10.00	
**On how many days in the past 7 days did your symptoms cause you to miss school or work or leave you unable to carry out your normal daily responsibilities?**				
*n*	1,471	1,042	429	MW: <0.0001
Median	0.00	0.00	1.00	
Q1	0.00	0.00	0.00	
Q3	3.00	2.00	4.00	
IQR	3.00	2.00	4.00	
**On how many days in the past 7 days did you feel so impaired by your symptoms that, even though you went to school or work or had other daily responsibilities, your productivity was reduced?**				
*n*	1,472	1,043	429	MW: 0.0693
Median	2.00	2.00	2.00	
Q1	0.00	0.00	0.00	
Q3	4.00	3.00	4.00	
IQR	4.00	3.00	4.00	

*SDS, Sheehan Disability Scale; MDD, major depressive disorder; TRD, treatment-resistant depression; MW, Mann-Whitney non-parametric test; Q, quartile; IQR, interquartile range*.

* *Scale value key: 0, not at all; 1–3, mildly; 4–6, moderately; 7–9, markedly; 10, extremely*.

## Discussion

As presented in the primary interim analysis report, among all MDD patients in this study, 29% had TRD. In the current report, we present a deeper exploration of factors relating to MDD and TRD in Latin American countries, including comorbidities, suicidality, and HRQoL. Common comorbidities differentiating TRD patients from non-TRD patients included GAD, PTSD, and substance use disorder (non-alcohol).

TRD patients had higher suicidal ideation and behavior scores across scales than did non-TRD patients, although suicidality substantially contributed to disease burden in both TRD and non-TRD. These rates are comparable to those found in a systematic review of TRD in the United States, which showed 15% of TRD patients reported suicidal ideation (compared with 1% in the general population) and 17% of patients had a history of suicide attempt (17).

For all measures presented, TRD was associated with a higher risk of suicidal behavior vs. non-TRD. This was demonstrated in results from the current disease status questionnaire, the MINI, and the C-SSRS, which is considered a complete instrument encompassing several dimensions of suicidal behavior. More than 90% of TRD patients reported feeling hopeless or pessimistic, and approximately 80% had feelings of guilt, worthlessness, or helplessness (vs. three-quarters of non-TRD patients in both cases), indicating a significantly increased disease burden for TRD patients. Compared with non-TRD patients, TRD patients were significantly more likely to report a “wish to be dead” (Figure 1), tended to have a greater degree of seriousness associated with suicidal thoughts/suicidality as well as significantly more intense suicidal ideation, as indicated by the most severe ideation, frequency of thoughts, and controllability of those thoughts on the C-SSRS (Figure 2). TRD patients were also more likely to display suicidal behavior. Significantly greater proportions of TRD patients had active suicidal behavior disorder per the MINI, answered “yes” to at least 1 of the C-SSRS suicidal behavior questions, and were significantly more likely to have had lifetime suicidal behavior, with nearly 3 in 10 reporting a lifetime attempt.

Although the burden of suicidality was greater for TRD patients, non-TRD patients reported suicidal ideation and behavior in substantial numbers, indicating that suicidality is a significant problem among all MDD patients in Latin America. According to the World Health Organization, regional age-standardized suicide rates per 100,000 individuals in 2012 were 4.2 in Mexico, 5.4 in Colombia, 10.3 in Argentina, and 5.8 in Brazil (3). Differences among countries may be accounted for by societal and cultural risk factors, including health care access barriers, access to means of suicide, inappropriate media reporting and social media use, and stigma associated with help-seeking behavior. These risk factors for suicidality among TRD patients in Latin America underscore the need for appropriate management of TRD to ameliorate disease burden and potentially increase survival.

TRD also significantly impacted HRQoL, consistent with previous studies in TRD patients that demonstrated a negative impact of TRD on mental and physical well-being; TRD was also associated with greater work impairments and productivity loss, both in the primary interim analysis of this study and in other analyses (18, 19, 21). A previous study reported that worse HRQoL was associated with higher suicidal ideation in MDD patients (26). In the present study, the EQ-5D-5L questionnaire revealed that patients with TRD were more likely to have problems walking, washing, dressing, or doing usual activities, and experienced more pain or discomfort and feelings of anxiety or depression; TRD patients also perceived themselves as being in significantly worse health than did non-TRD patients.

In our study, the difference in painful symptoms between the TRD and non-TRD groups was notable; more than twice the number of TRD patients (23.5%) reported severe or extreme pain or discomfort compared with non-TRD patients. Physical pain has been shown to be an important risk factor for suicide, pointing to a possible association with the relationship between treatment resistance and suicidal behavior (27, 28).

Results from the PHQ-9 demonstrated that TRD patients reported more severe depression than non-TRD patients and that this depression more severely impacted everyday activities and relationships; over two-thirds of TRD patients had problems that made it very or extremely difficult to work, take care of tasks at home, or get along with others in comparison with less than half of non-TRD patients. These results are comparable to those of the MADRS instrument employed in the primary interim analysis, which reported significantly more TRD patients with severe depression (MADRS total score >34) compared with non-TRD patients (21).

Responses to the SDS also showed that TRD patients, vs. non-TRD patients, were disproportionally disrupted in multiple functional domains, including work/school, social life/leisure activities, and family life/home responsibilities, and experienced more days in the previous week in which they found it impossible to carry out normal daily responsibilities. While there can be some debate as to whether HRQoL measures or symptomatic measures are better indicators of disease burden, results from this secondary interim analysis of the TRAL study showed that TRD patients in the Latin American countries studied are disproportionally impacted in both domains.

Based on the findings of this study, in which the severity of depression was greater in patients with TRD compared with non-TRD, clinicians in daily clinical practice could be more attentive in recognizing and diagnosing TRD in patients with severe depression. This is especially important in health care systems (in this case, those in Latin American countries) in which a practitioner may see a patient only once without the possibility of monitoring the same patient in the medium term. To this point, follow-up is a primary challenge in treating patients with TRD. Improving patient access to follow-up care as well as to continuity of care may have a positive impact on the quality of treatment, costs, and HRQoL for TRD patients, a group disproportionally affected by the potential for suicidality and detriments to daily activities, ranging from basic self-care to the ability to work and maintain relationships with others. In addition, new treatment options for TRD are needed to potentially improve outcomes in this underserved patient population.

A limitation of the present analysis is the lack of country-specific information about suicidality; country-based analyses of these data are currently in progress. Also, the current work represents an interim analysis; further information will be reported when the final analyses are complete.

Critically, TRAL is not a population-based study; patients enrolled in the study were recruited through regional centers that provide treatment and services for those with mental disorders (e.g., clinics and hospitals, as opposed to specialty treatment centers). This is an important caveat in a depression prevalence study, as many cases of depression may not be diagnosed in a general medicine practice. Diagnosis of MDD using the MINI, rather than presumptive diagnoses from patient registry databases, is a strength of this study. MDD may be underdiagnosed, but analysis of those with this diagnosis may provide important information about the differences between TRD patients and non-TRD patients.

Additionally, this study enrolled patients with a new or continued episode of depression, regardless of whether that episode was treated or untreated. This may have introduced a complicating factor into the current analysis, as those who perceive that they have an unmet need for mental health services (i.e., those who are un- or undertreated) are more likely to report suicidal ideation and behavior (29). However, only 11 out of 429 TRD patients were untreated at the time of enrollment (21), suggesting a minimal impact on the differences in suicidality between TRD and non-TRD patients.

## Conclusions

TRD patients are more likely than non-TRD patients to experience suicidality and detriments to HRQoL. TRD represents a substantial unmet treatment need among MDD patients in Latin America.

## Data Availability Statement

The data sharing policy of Janssen Pharmaceutical Companies of Johnson & Johnson is available at https://www.janssen.com/clinical-trials/transparency. Requests to access the datasets should be directed to the Yale Open Data Access (YODA) Project site at http://yoda.yale.edu.

## Ethics Statement

The studies involving human participants were reviewed and approved by CEP HUPES, Comitê de Ética em Pesquisa do Hospital Universitário Walter Cantídio, COEP-UFMG, CEP- INC—Instituto de neurologia de Curitiba, Comite de Etica em pesquisa IPUB-UFRJ, Comitê de Ética em Pesquisa Investiga—Institutos de Pesquisa, Comitê de Ética em Pesquisa em Seres Humanos da Faculdade de Medicina da Universidade Federal de Pelotas, Comissão de Ética para Análise de Projetos de Pesquisa—CAPPesq, Comitê de Ética em Pesquisa Hospital São José, Comitê de Ética em Pesquisado Hospital de Clínicas de Porto Alegre (CEP/HCPA), C.E.I. Campo Abierto LTDA, E.S.E. Hospital Mental de Antioquia, Clínica CEIC de la Fundación Centro de Investigación Clínica, CIC, Comité Independiente de Ética de Investigación en Salud Prof. Dr. Marcelino Rusculleda, Comité de Bioética e Investigación de la Fundación para el Estudio y Tratamiento de las Enfermedades Mentales (FETEM), AICI—CIAP, Instituto Centralizado de Asistencia e Investigación Clínica Integral—Centro de Investigación y Asistencia en Psiquiatría, Instituto Médico Platense S.A., Comité Independiente de Ética para Ensayo en Farmacología Clínica. Fundación de Estudios Farmacológicos y de Medicamentos Prof. Luis M. Zeiher, Sanatorio Alcocer Pozo S.A. de C.V., Investigación Biomédica para el Desarrollo de Fármacos, S.A. de C.V., Instituto Nacional de Neurología y Neurocirugía Manuel Velazco Suárez, Hospital La Misión, S.A. de C.V., Instituto Nacional de Psiquiatría Ramón de la Fuente Muñiz, Hospital Central Dr. Ignacio Morones Prieto, and Comité Institucional de Ética en Investigación- ISSSTE. The patients/participants provided their written informed consent to participate in this study.

## Author Contributions

All authors meet the ICMJE authorship criteria, giving substantial contribution to the conception or design of the work, data acquisition and analysis, drafting or reviewing the work for intellectual content, and giving final approval of the version to be published. The authors agreed to be accountable for all aspects of the work in ensuring that questions related to the accuracy or integrity of any part of the work are appropriately investigated and resolved.

## Funding

This study received funding from Janssen Pharmaceutical Companies of Johnson & Johnson. The funder had the following involvement with the study: study design, conduction, monitoring, database analysis, medical writing of the manuscript, and submission for publication.

## Conflict of Interest

RC has received funding and honoraria from Janssen for research and serving in an advisory role and as a speaker. HA has received research funding from, and served as a speaker for, Janssen-Cilag. LMAB has received research funding from Janssen-Cilag, Lundbeck, Acadia, TEVA Pharmaceuticals, Otsuka, and Takeda. EF, XD, and MAC have received research funding from Janssen. LQ has received consulting fees from Allergan, Abbot, Cristalia, Janssen Pharmaceutical, and Lundbeck, and has received research funding from Fapex and Janssen Pharmaceutical. PC was an employee of Janssen-Cilag at the time of her work on this study and is currently affiliated with Janssen Global Services, Inc. GK is an employee of Janssen-Cilag. This study received funding from Janssen Pharmaceutical Companies of Johnson & Johnson. The funder had the following involvement with the study: study design, conduction, monitoring, database analysis, medical writing of the manuscript, and submission for publication.

## Publisher's Note

All claims expressed in this article are solely those of the authors and do not necessarily represent those of their affiliated organizations, or those of the publisher, the editors and the reviewers. Any product that may be evaluated in this article, or claim that may be made by its manufacturer, is not guaranteed or endorsed by the publisher.
